# 2-Methyl­imidazolium hydrogen maleate

**DOI:** 10.1107/S1600536809004103

**Published:** 2009-02-11

**Authors:** Zhi-Xiong Liu

**Affiliations:** a College of Horticulture and Gardening, Yangtze University, Jingzhou 434025, Hubei, People’s Republic of China, and College of Biological Science and Biotechnology, Beijing Forestry University, Beijing 100083, People’s Republic of China

## Abstract

Mol­ecules in the title compound, C_4_H_7_N_2_^+^·C_4_H_3_O_4_^−^, are linked by inter­molecular N—H⋯O hydrogen bonds into one-dimensional chains parallel to [101]. These chains are in turn linked by an *R*_2_^2^(8) motif, formed by weak C—H⋯O hydrogen bonds, into corrugated sheets running parallel to (10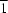). These sheets are further linked by weak inter­molecular C—H⋯O hydrogen bonds, forming a three-dimensional network. Intra­molecular N—H⋯O and O—H⋯O inter­actions are also present.

## Related literature

For related structures, see: Aakeröy & Salmon (2005 ▶); Liu & Meng (2006 ▶). For hydrogen-bond motifs, see: Bernstein *et al.* (1995 ▶).
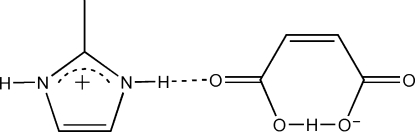


## Experimental

### 

#### Crystal data

C_4_H_7_N_2_^+^·C_4_H_3_O_4_^−^
*M* *_r_* = 198.18
Monoclinic, 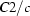

*a* = 13.9897 (14) Å
*b* = 7.2274 (7) Å
*c* = 20.533 (2) Å
β = 108.310 (2)°
*V* = 1970.9 (3) Å^3^
*Z* = 8
Mo *K*α radiation
μ = 0.11 mm^−1^
*T* = 295 (2) K
0.10 × 0.10 × 0.08 mm

#### Data collection

Bruker SMART APEX CCD area-detector diffractometer
Absorption correction: multi-scan (*SADABS*; Sheldrick, 2001 ▶) *T*_min_ = 0.979, *T*_max_ = 0.991
7461 measured reflections
2143 independent reflections
1273 reflections with *I* > 2σ(*I*)
*R*_int_ = 0.027

#### Refinement

*R*[*F* ^2^ > 2σ(*F* ^2^)] = 0.046
*wR*(*F* ^2^) = 0.130
*S* = 0.99
2143 reflections
137 parameters
H atoms treated by a mixture of independent and constrained refinement
Δρ_max_ = 0.17 e Å^−3^
Δρ_min_ = −0.13 e Å^−3^

### 

Data collection: *SMART* (Bruker, 2001 ▶); cell refinement: *SAINT-Plus* (Bruker, 2001 ▶); data reduction: *SAINT-Plus*; program(s) used to solve structure: *SHELXS97* (Sheldrick, 2008 ▶); program(s) used to refine structure: *SHELXL97* (Sheldrick, 2008 ▶); molecular graphics: *PLATON* (Spek, 2003 ▶); software used to prepare material for publication: *PLATON*.

## Supplementary Material

Crystal structure: contains datablocks global, I. DOI: 10.1107/S1600536809004103/lh2769sup1.cif

Structure factors: contains datablocks I. DOI: 10.1107/S1600536809004103/lh2769Isup2.hkl

Additional supplementary materials:  crystallographic information; 3D view; checkCIF report

## Figures and Tables

**Table 1:** Hydrogen-bond geometry (Å, °)

*D*—H⋯*A*	*D*—H	H⋯*A*	*D*⋯*A*	*D*—H⋯*A*
O1—H3*A*⋯O3	1.20 (3)	1.21 (3)	2.4085 (18)	174 (2)
N1—H1⋯O2	0.901 (19)	1.80 (2)	2.701 (2)	176.3 (17)
N2—H2*A*⋯O4^i^	0.95 (2)	1.77 (2)	2.713 (2)	171.2 (17)
C3—H3⋯O3^ii^	0.93	2.64	3.471 (2)	150
C4—H4*A*⋯O3^i^	0.96	2.59	3.490 (2)	155
C6—H6⋯O2^iii^	0.93	2.66	3.544 (2)	158

Symmetry codes: (i) 
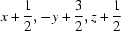
; (ii) 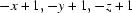; (iii) 
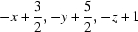
.

**Fig. 1.**
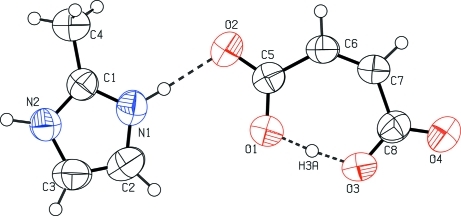
Molecular structure of (I), showing the atom-numbering scheme. Displacement ellipsoids are drawn at the 50% probability level. H-bonds are shown in dashed lines.

**Fig. 2.**
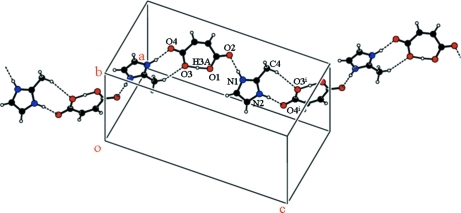
Part of the crystal structure of (I), showing the formation of the one-dimensional chain linked by intermolecular N-H···O hydrogen bonds parallel to the [101] direction. (symmetry code: i = 1/2+x, 3/2-y, 1/2+z)

**Fig. 3.**
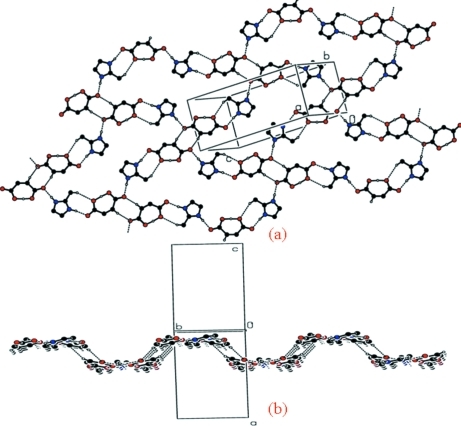
Part of the crystal structure of (I), showing the formation of the two-dimensional corrugated sheet linked by intermolecular N-H···O and C-H···O hydrogen bonds (a) view perpendicular to the (101) plane and (b) view along to the (101) plane, respectively. Only H atoms involved in hydrogen bonds are shown.

## supplementary crystallographic information

### Comment

As part of the continuing studies on the synthesis of co-crystal or organic salts involving imidazole (Liu & Meng, 2006), the crystal structure of title compound (I) is reported. It was obtained by mixing a 2:1 molar amounts of 2-methylimidazole and 2-maleic acid and in 95% methanol solution at room temperature.

According to Aakeröy and Salmon (2005) complex (I) is an organic salt. In (I), one of the carboxyl protons is transferred to the imidazole N atom, forming a 1:1 anhydrous organic adduct. The two carboxyl groups in the maleate anion are hydrogen-bonded to each other via atom H3A located approximately at the mid-point of atoms O1 and O3 (Fig.1).

In the crystal structure, by a combination of N1-H1···O2, N2-H2A···O4^i^ and C4-H4A···O3^i^ hydrogen bonds (symmetry codes as in Table 1) molecules in (I) are linked into a one-dimensional chain parallel to the [101] direction (Fig.2). These adjacent chains are linked by a *R*_2_^2^(8) hydrogen motif (Bernstein *et al.*,  1995) originating from two weak centrosymmetric C6-H6···O2 (3/2-*x*, 5/2-*y*, 1-*z*) hydrogen bonds, into a corrugated sheet running parallel to the (101) plane (Fig.3). These sheets are further linked by weak C3-H3···O3 (1-*x*, 1-*y*, 1-*z*) hydrogen bonds, forming a three-dimensional network.

### Experimental

All the reagents and solvents were used as obtained without further purification. A 1:2 molar amounts of maleic acid (0.1 mmol, 11.6 mg) and 2-methylimidazole (0.2 mmol, 16.4 mg) were dissolved in 95% methanol (10 ml). The mixture was stirred for half an hour at room temperature and then filtered. The resulting solution was kept in air for one week. Block-shaped crystals suitable for single-crystal X-ray diffraction analysis were grown by slow evaporation of a solution of (I).

### Refinement

H atoms bonded to C atoms were located in difference maps and subsequently treated as riding modes, with C–H=0.93 Å, *U*_iso_(H) = 1.2*U*_eq_(C) and C–H=0.96 Å, 1.5*U*_eq_(C) for methyl H atoms. H atoms bonded to N and O atoms were also found in the difference maps and their distances were refined freely (see Table 1 for the distances), and the *U*_iso_(H) values being set k times of their carrier atoms (k=1.2 for N and 1.5 for O atoms)

### Crystal data

**Table:**

C_4_H_7_N_2_^+^·C_4_H_3_O_4_^−^	*F*(000) = 832
*M**_r_* = 198.18	*D*_x_ = 1.336 Mg m^−^^3^
Monoclinic, *C*2/*c*	Mo *K*α radiation, λ = 0.71073 Å
Hall symbol: -C 2yc	Cell parameters from 1457 reflections
*a* = 13.9897 (14) Å	θ = 3.1–21.4°
*b* = 7.2274 (7) Å	µ = 0.11 mm^−^^1^
*c* = 20.533 (2) Å	*T* = 295 K
β = 108.310 (2)°	Block, colorless
*V* = 1970.9 (3) Å^3^	0.10 × 0.10 × 0.08 mm
*Z* = 8	

### Data collection

**Table:**

Bruker SMART APEX CCD area-detector diffractometer	2143 independent reflections
Radiation source: fine focus sealed Siemens Mo tube	1273 reflections with *I* > 2σ(*I*)
graphite	*R*_int_ = 0.027
0.3° wide ω exposures scans	θ_max_ = 27.0°, θ_min_ = 2.1°
Absorption correction: multi-scan (*SADABS*; Sheldrick, 2001)	*h* = −17→17
*T*_min_ = 0.979, *T*_max_ = 0.991	*k* = −9→9
7461 measured reflections	*l* = −26→24

### Refinement

**Table:**

Refinement on *F*^2^	Primary atom site location: structure-invariant direct methods
Least-squares matrix: full	Secondary atom site location: difference Fourier map
*R*[*F*^2^ > 2σ(*F*^2^)] = 0.046	Hydrogen site location: inferred from neighbouring sites
*wR*(*F*^2^) = 0.130	H atoms treated by a mixture of independent and constrained refinement
*S* = 0.99	*w* = 1/[σ^2^(*F*_o_^2^) + (0.0703*P*)^2^] where *P* = (*F*_o_^2^ + 2*F*_c_^2^)/3
2143 reflections	(Δ/σ)_max_ = 0.001
137 parameters	Δρ_max_ = 0.17 e Å^−^^3^
0 restraints	Δρ_min_ = −0.13 e Å^−^^3^

### Special details

**Table:**

Geometry. All esds (except the esd in the dihedral angle between two l.s. planes) are estimated using the full covariance matrix. The cell esds are taken into account individually in the estimation of esds in distances, angles and torsion angles; correlations between esds in cell parameters are only used when they are defined by crystal symmetry. An approximate (isotropic) treatment of cell esds is used for estimating esds involving l.s. planes.
Refinement. Refinement of F^2^ against ALL reflections. The weighted R-factor wR and goodness of fit S are based on F^2^, conventional R-factors R are based on F, with F set to zero for negative F^2^. The threshold expression of F^2^ > 2sigma(F^2^) is used only for calculating R-factors(gt) etc. and is not relevant to the choice of reflections for refinement. R-factors based on F^2^ are statistically about twice as large as those based on F, and R- factors based on ALL data will be even larger.

### Fractional atomic coordinates and isotropic or equivalent isotropic displacement parameters (Å^2^)

**Table:**

	*x*	*y*	*z*	*U*_iso_*/*U*_eq_	
C1	0.77143 (12)	0.6738 (2)	0.65115 (8)	0.0603 (4)	
C2	0.64878 (15)	0.5430 (3)	0.57064 (11)	0.0810 (6)	
H2	0.5957	0.5264	0.5303	0.097*	
C3	0.68665 (14)	0.4163 (3)	0.61863 (11)	0.0812 (6)	
H3	0.6654	0.2943	0.6183	0.097*	
C4	0.84501 (15)	0.8105 (3)	0.69038 (10)	0.0855 (6)	
H4A	0.8699	0.7731	0.7376	0.128*	
H4B	0.8131	0.9293	0.6872	0.128*	
H4C	0.9000	0.8183	0.6720	0.128*	
C5	0.61393 (13)	1.0269 (2)	0.46763 (9)	0.0630 (5)	
C6	0.59800 (13)	1.1982 (2)	0.42603 (9)	0.0641 (5)	
H6	0.6446	1.2915	0.4440	0.077*	
C7	0.52819 (11)	1.2406 (2)	0.36726 (9)	0.0643 (5)	
H7	0.5327	1.3601	0.3517	0.077*	
C8	0.44457 (13)	1.1297 (3)	0.32255 (9)	0.0652 (5)	
N1	0.70226 (11)	0.7022 (2)	0.59149 (8)	0.0687 (4)	
H1	0.6924 (14)	0.811 (3)	0.5686 (9)	0.082*	
N2	0.76299 (11)	0.4998 (2)	0.66877 (8)	0.0665 (4)	
H2A	0.8074 (14)	0.439 (3)	0.7076 (10)	0.080*	
O1	0.55899 (10)	0.88414 (17)	0.44508 (7)	0.0830 (4)	
O2	0.68111 (10)	1.02685 (18)	0.52321 (7)	0.0844 (4)	
O3	0.43419 (11)	0.96097 (18)	0.33767 (7)	0.0885 (5)	
H3A	0.495 (2)	0.916 (3)	0.3917 (15)	0.133*	
O4	0.38675 (9)	1.20541 (19)	0.27165 (6)	0.0826 (4)	

### Atomic displacement parameters (Å^2^)

**Table:**

	*U*^11^	*U*^22^	*U*^33^	*U*^12^	*U*^13^	*U*^23^
C1	0.0574 (10)	0.0627 (11)	0.0610 (10)	0.0021 (8)	0.0187 (8)	−0.0008 (8)
C2	0.0663 (11)	0.0887 (15)	0.0787 (13)	−0.0016 (10)	0.0093 (10)	−0.0184 (11)
C3	0.0727 (12)	0.0667 (12)	0.0994 (15)	−0.0082 (10)	0.0202 (11)	−0.0139 (11)
C4	0.0861 (13)	0.0770 (14)	0.0872 (14)	−0.0151 (10)	0.0186 (11)	−0.0040 (10)
C5	0.0611 (10)	0.0644 (11)	0.0618 (11)	−0.0024 (8)	0.0169 (9)	−0.0047 (8)
C6	0.0662 (10)	0.0583 (10)	0.0644 (11)	−0.0138 (8)	0.0158 (9)	−0.0052 (8)
C7	0.0649 (10)	0.0580 (10)	0.0660 (11)	−0.0100 (8)	0.0147 (9)	−0.0006 (8)
C8	0.0631 (11)	0.0734 (12)	0.0588 (11)	−0.0047 (9)	0.0188 (9)	−0.0052 (9)
N1	0.0679 (9)	0.0715 (10)	0.0631 (9)	0.0079 (8)	0.0154 (8)	0.0042 (7)
N2	0.0633 (9)	0.0610 (9)	0.0712 (10)	0.0041 (7)	0.0156 (8)	0.0035 (7)
O1	0.0929 (9)	0.0625 (8)	0.0787 (9)	−0.0138 (7)	0.0056 (8)	0.0039 (6)
O2	0.0830 (9)	0.0878 (10)	0.0658 (8)	−0.0080 (7)	−0.0005 (7)	0.0060 (6)
O3	0.0962 (10)	0.0716 (9)	0.0768 (9)	−0.0281 (7)	−0.0028 (7)	−0.0017 (7)
O4	0.0748 (8)	0.0894 (9)	0.0693 (8)	−0.0015 (7)	0.0021 (7)	0.0008 (7)

### Geometric parameters (Å, °)

**Table:**

C1—N1	1.317 (2)	C5—O1	1.283 (2)
C1—N2	1.324 (2)	C5—C6	1.481 (2)
C1—C4	1.471 (2)	C6—C7	1.328 (2)
C2—C3	1.327 (3)	C6—H6	0.9300
C2—N1	1.366 (2)	C7—C8	1.476 (2)
C2—H2	0.9300	C7—H7	0.9300
C3—N2	1.369 (2)	C8—O4	1.2307 (19)
C3—H3	0.9300	C8—O3	1.278 (2)
C4—H4A	0.9600	N1—H1	0.901 (19)
C4—H4B	0.9600	N2—H2A	0.95 (2)
C4—H4C	0.9600	O1—H3A	1.20 (3)
C5—O2	1.230 (2)	O3—H3A	1.21 (3)
			
N1—C1—N2	107.46 (16)	C7—C6—C5	130.67 (15)
N1—C1—C4	125.99 (16)	C7—C6—H6	114.7
N2—C1—C4	126.55 (16)	C5—C6—H6	114.7
C3—C2—N1	107.26 (17)	C6—C7—C8	130.74 (16)
C3—C2—H2	126.4	C6—C7—H7	114.6
N1—C2—H2	126.4	C8—C7—H7	114.6
C2—C3—N2	106.84 (18)	O4—C8—O3	122.41 (17)
C2—C3—H3	126.6	O4—C8—C7	117.90 (17)
N2—C3—H3	126.6	O3—C8—C7	119.69 (16)
C1—C4—H4A	109.5	C1—N1—C2	109.26 (16)
C1—C4—H4B	109.5	C1—N1—H1	124.6 (12)
H4A—C4—H4B	109.5	C2—N1—H1	126.1 (12)
C1—C4—H4C	109.5	C1—N2—C3	109.18 (16)
H4A—C4—H4C	109.5	C1—N2—H2A	125.4 (11)
H4B—C4—H4C	109.5	C3—N2—H2A	125.1 (11)
O2—C5—O1	122.12 (17)	C5—O1—H3A	111.7 (11)
O2—C5—C6	117.90 (15)	C5—O2—H1	113.4 (6)
O1—C5—C6	119.98 (16)	C8—O3—H3A	112.4 (11)
			
N1—C2—C3—N2	0.3 (2)	C4—C1—N1—C2	179.34 (16)
O2—C5—C6—C7	−175.16 (18)	C3—C2—N1—C1	−0.1 (2)
O1—C5—C6—C7	5.4 (3)	N1—C1—N2—C3	0.27 (19)
C5—C6—C7—C8	−1.9 (3)	C4—C1—N2—C3	−179.18 (16)
C6—C7—C8—O4	176.67 (17)	C2—C3—N2—C1	−0.3 (2)
C6—C7—C8—O3	−3.0 (3)	O1—C5—O2—H1	−0.7 (7)
N2—C1—N1—C2	−0.11 (19)	C6—C5—O2—H1	179.9 (6)

### Hydrogen-bond geometry (Å, °)

**Table:**

*D*—H···*A*	*D*—H	H···*A*	*D*···*A*	*D*—H···*A*
O1—H3A···O3	1.20 (3)	1.21 (3)	2.4085 (18)	174 (2)
N1—H1···O2	0.901 (19)	1.80 (2)	2.701 (2)	176.3 (17)
N2—H2A···O4^i^	0.95 (2)	1.77 (2)	2.713 (2)	171.2 (17)
C3—H3···O3^ii^	0.93	2.64	3.471 (2)	150
C4—H4A···O3^i^	0.96	2.59	3.490 (2)	155
C6—H6···O2^iii^	0.93	2.66	3.544 (2)	158

Symmetry codes: (i) *x*+1/2, −*y*+3/2, *z*+1/2; (ii) −*x*+1, −*y*+1, −*z*+1; (iii) −*x*+3/2, −*y*+5/2, −*z*+1.
